# Vaginal douching in Zambia: a risk or benefit to women in the fight against cervical cancer: a retrospective cohort study

**DOI:** 10.1186/s12905-019-0834-y

**Published:** 2019-11-09

**Authors:** Twaambo Euphemia Hamoonga, Pawel Olowski, Patrick Musonda

**Affiliations:** 1 Department of Community and Family Medicine, Population Studies Unit, School of Public Health, University of Zambia, P O Box 50110, Lusaka, Zambia; 2 Department of Epidemiology & Biostatistics, School of Public Health, University of Zambia, Lusaka, Zambia

**Keywords:** Cervical cancer, Risk, Benefit, Abnormal cervical lesions, Douching, Zambia, Women

## Abstract

**Background:**

Cervical cancer was the most commonly diagnosed cancer and the leading cause of cancer related deaths in 2013 among women in Zambia. We determined factors associated with vaginal douching with any solution other than water and examined its role as a risk factor for abnormal cervical lesions among Zambian women.

**Methods:**

We conducted a retrospective cohort study using data from the Cervical Cancer Prevention Program in Zambia among 11,853 women (15 years or older) who had screened for cervical cancer from 6 provinces of Zambia. Stata version 15 was used to analyze the data. Investigator led stepwise logistic regression was used to estimate adjusted odds ratios and 95% confidence intervals for various characteristics, with vaginal douching with any solution as primary outcome and abnormal cervical lesions as secondary outcome.

**Results:**

Douching with any solution other than water was practiced by 8.1% (*n* = 960) of the study participants. Older women (35–44 and 45 years or older) vs young women (15–24 years old) were less likely to douche with a solution (AOR 0.74; 95% CI: 0.57–0.97, *p* = 0.027 and AOR 0.65; 95% CI: 0.49–0.87, *P* = 0.004), respectively, and so were women in informal employment compared to housewives (AOR 0.72; 95% CI: 0.58–0.89, *p* = 0.002). Odds of douching were higher among women with secondary vs. no formal education (AOR 1.64; 95% CI: 1.15–2.35, *P* = 0.007), and among women who used condoms sometimes compared to those who never with their regular sexual partners (AOR 1.19; 95% CI: 1.01–1.40, PP = 0.037). About 12.2% of study participants had abnormal cervical lesions. The use of either vinegar, ginger, lemon, salt or sugar solution was associated with increased risk of abnormal cervical lesions (AOR 7.37; 95% CI: 1.43–38.00, *p* = 0.017) compared to using water.

**Conclusion:**

We find an association between douching with a solution and a woman’s age, educational attainment, occupation and condom use. Vaginal douching with either vinegar, ginger, lemon, salt or sugar solution was associated with increased risk for abnormal cervical lesions. We recommend further research on ever vs never douching and the risk for abnormal cervical lesions.

## Background

Cancer is an emerging public health problem in Africa [1]. According to the GLOBOCAN 2018 estimates, the share of cancer deaths in Africa (7.3%) is higher than the share of incidence (5.8%) [2]. Cervical cancer ranks second in incidence and mortality behind breast cancer in lower human development index (HDI) settings, with Africa recording the highest regional incidence and mortality rates [2]. Zambia, Malawi, Mozambique, and Tanzania have among the highest cervical cancer rates (50 cases per 100,000) worldwide [3]. In Zambia, cervical cancer was the most commonly diagnosed cancer and the leading cause of cancer related deaths in 2013 among women [4]. The mortality rate from the disease could be attributed, in part, to the fact that most cases (about 80%) are advanced at presentation, when only palliative treatment can be given [5].

The cause of cervical cancer has been postulated to be multifactorial including behavioral factors such as vaginal douching. Vaginal douching is the process of intravaginal cleansing with a liquid solution [6]. It is used for personal hygiene or aesthetic reasons, for preventing or treating an infection [7], to cleanse after menstruation or sex, and to prevent pregnancy [8]. For example, alum, an astringent, was used for various purposes such as tightening of the vagina for enhancement of sexual pleasure, making the vagina ‘younger’, or to hide evidence of infidelity [9]. Another common practice is that associated with dry sex, where individuals prefer a dry, tight vagina during sexual intercourse [10]. Dry sex more often than not involves the use of plants to dry and contract the vagina, a popular practice in Africa that damages vaginal tissue and facilitates the spread of sexually transmitted diseases [11, 12].

Very few studies have examined the association between vaginal douching and abnormal cervical lesions. Studies that have examined the association have conflicting views on the benefits or harm associated with douching [6]. Nevertheless, most studies have hypothesized that frequent douching alters the vaginal chemical environment, making the cervix more susceptible to pathologic change, and serious gynecologic outcomes, including increased risk of cervical cancer, pelvic inflammatory disease, endometritis, and increased risk for sexually transmitted infections, including HIV [13–16]. We determined factors associated with douching with any solution other than water. We also examined the association between abnormal cervical lesions and douching with such solutions among Zambian women.

## Methods

### Study design and setting

A quantitative retrospective cohort study was conducted in order to determine factors associated with douching with any solution (as primary outcome) and to examine the association between using these solutions and risk for abnormal cervical lesions (being a secondary outcome). This study was conducted at the University Teaching Hospital’s Centre for Infectious Disease Research in Zambia (CIDRZ) using programmatic data from the Cervical Cancer Prevention Program in Zambia (CCPPZ). Details on the CCPPZ are explained in our previous publication [17]. Briefly, the CCPPZ is a program that was launched in 2006 to increase access to cervical cancer screening in the quest to reduce the incidence and prevalence of the disease. Through this program, cervical cancer screening services are freely available at most of the public health facilities across the country. All women who are, and have been sexually active, can freely walk into any of the facilities offering screening services and get screened for cervical cancer. Cervical cancer screening is done using visual inspection with dilute (5%) acetic acid (VIA) linked to immediate cryotherapy (see and treat). Prior to screening, self-reported data (socio-demographics, sexual behaviour, and other medical related history) is captured electronically for each woman seeking screening services. VIA test results are also recorded for each woman screened.

### Data extraction

A data extraction sheet was used to extract data for 11,853 women aged 15 years or older who had ever screened for cervical cancer at various public health facilities in 6 provinces of Zambia. To be eligible to participate in this study, women needed to have had at least one sexual partner in their lifetime. Women whose records had incomplete information on HIV status, type of douche used and the VIA test results were excluded from the study. For HIV status, women who indicated that they did not know their HIV status were included in the study. However, women with missing data (neither positive, negative nor unknown) were excluded from the study. For the purpose of this study, douching with any solution was defined as any act involving the introduction of any solution other than water, into the vagina. A VIA positive result was indicative of an abnormal cervical lesion, where an abnormal cervical lesion was defined as an aceto-white lesion or whitish patch on the uterine cervix when ‘painted’ or ‘stained’ with 5% acetic-acid vinegar [18].

### Data analysis

For data analysis, vaginal douching with any solution other than water was the primary outcome while abnormal cervical lesion was the secondary outcome. The socio-demographic and sexual behavior characteristics were the predictor variables. The data that was extracted from the CCPPZ database was entered in excel and exported to Stata version 15 where both descriptive and analytical methods of data analysis were used. Descriptive statistics were used to obtain numbers and proportions of women by their socio-demographic characteristics. The chi-square test of association was used to determine associations between douching with any solution and the various socio-demographic and sexual behavioral characteristics of participants. We used logistic regression analysis to determine the predictors of douching with any solution. Secondary analysis was also conducted to identify types of douches that were risk factors for abnormal cervical lesions. We used a significance level of 10% for independent variables to be entered in the multivariable analysis and the overall significance level in the adjusted model was taken to be the traditional 5%. AORs, *p*-values and the associated 95% confidence intervals (CIs) were estimated and used as measures of effect.

### Ethics

We obtained ethical approval to conduct this study from the Research Ethics and Science Converge committee (ERES) in Zambia. Permission to use the CCPPZ data was obtained from the Director- CIDRZ. This being programmatic data, no consent was obtained from study participants, however, we ensured that all identifiers were removed from the dataset to guarantee anonymity of study participants.

## Results

### Social demographic characteristics

This study was conducted among 11,853 women who had ever screened for cervical cancer from various health facilities in six provinces of Zambia between 2006 and 2014. The prevalence of douching with a solution other than water was 8.1% (*n* = 960). The rest of the women (91.9%) douched with water. Table 1 shows the association between douching and women’s socio-demographic and sexual behavioral characteristics. Douching with any solution was associated with age (*p* = 0.009), educational attainment (*p* = 0.004), occupation (*p* = 0.001), number of life time sexual partners (*p* = 0.005) and condom use with regular sexual partner (*p* < 0.001). Among women who douched with any solution, the largest proportion were aged between 25 and 34 years (35.7%), had 2–5 sexual partners (66%) and had attained secondary education (41.1%). About 45% were housewives and slightly more than half of them (51.2%) never used condoms with their regular sexual partner(s).

**Table 1:** Frequency distribution and chi-square test of association for douching with any solution other than water

	Douching with any solution		
Characteristic	Yes *n*(%)	No *n*(%)	*p*-value (chi^2^)
Total	960 (8.1)	10,983 (91.9)	
Age group			0.009
15–24	158 (16.7)	1446 (13.6)	
25–34	338 (35.7)	3661 (34.3)	
35–44	266 (28.1)	3108 (29.1)	
45+	185 (19.5)	2456 (23.0)	
Marital Status			0.428
Never married	120 (12.6)	1218 (11.3)	
Currently married	667 (70.2)	7605 (70.7)	
Widowed/separated/divorced	163 (17.2)	1938 (18.0)	
Education Level			0.004
No formal education	54 (5.7)	839 (7.8)	
Primary	337 (35.6)	4158 (38.6)	
Secondary	390 (41.1)	3877 (36.0)	
Tertiary	167 (17.6)	1908 (17.7)	
Occupation			0.001
Housewife	413 (45.0)	4503 (43.2)	
Formal employment	181 (19.7)	1709 (16.4)	
Informal employment	202 (22.0)	2881 (27.7)	
Other	121 (13.2)	1328 (12.7)	
Household income			0.495
Less than 100	13 (2.0)	114 (1.6)	
100–499	15 (2.3)	224 (3.1)	
500–999	38 (5.8)	440 (6.0)	
1000–5000	116 (17.7)	1157 (15.9)	
More than 5000	472 (72.2)	5359 (73.5)	
Age at sexual debut			0.568
< 20	726 (75.6)	8323 (76.4)	
20 years and older	234 (24.4)	2565 (23.6)	
Lifetime sexual partners			0.005
One partner	248 (26.1)	3370 (31.1)	
Two to five partners	628 (66.0)	6718 (62.0)	
More than five partners	75 (7.9)	748 (6.9)	
Condom use			< 0.001
Never	422 (51.2)	6051 (58.9)	
Sometimes	374 (45.4)	3886 (37.9)	
Always	28 (3.4)	329 (3.2)	
HIV status			0.105
Positive	218 (22.7)	2174 (20.0)	
Negative	555 (57.8)	6432 (59.0)	
Unknown	187 (19.5)	2287 (21.0)	

Table 2 presents results from both univariate and multivariable logistic regression analysis. Results from the univariate logistic regression analysis show that: age, education, occupation, number of life time sexual partners, condom use and HIV status were statistically associated with douching with a solution.

**Table 2:** Univariate and multivariable logistic regression analysis for factors associated with douching with any solution other than water

Characteristic	UOR (95% CI)	*p*-value	AOR (95% CI)	*p*-value
Age group (years)				
15–24	ref		ref	
25–34	0.84 (0.69–1.03)	0.096	0.82 (0.65–1.04)	0.109
35–44	0.78 (0.64–0.96)	0.02	0.74 (0.57–0.97)	0.027
45+	0.69 (0.55–0.86)	0.001	0.65 (0.49–0.87)	0.004
Educational attainment				
No formal education	ref		ref	
Primary	1.26 (0.94–1.69)	0.128	1.42 (0.99–2.02)	0.051
Secondary	1.56 (1.16–2.10)	0.003	1.64 (1.15–2.35)	0.007
Tertiary	1.36 (0.99–1.86	0.058	1.12 (0.73–1.72)	0.597
Marital status				
Never married	ref		ref	
Currently married	0.89 (0.72–1.09)	0.263	1.15 (0.86–1.53)	0.344
Widowed/separated/divorced	0.85 (0.67–1.09)	0.208	1.04 (0.75–1.44)	0.819
Occupation				
Housewife	ref		ref	
Formal employment	1.15 (0.96–1.39)	0.124	1.20 (0.91–1.58)	0.199
Informal employment	0.76 (0.64–0.91)	0.003	0.72 (0.58–0.89)	0.002
Other	0.99 (0.80–1.23)	0.951	1.00 (0.76–1.33)	0.969
Household income				
Less than 100	ref			
100–499	0.59 (0.27–1.28)	0.179		
500–999	0.76 (0.39–1.47)	0.411		
1000–5000	0.88 (0.48–1.61)	0.676		
More than 5000	0.77 (0.43–1.38)	0.384		
Age at sexual debut				
< 20 years	ref			
20 years or older	1.05 (0.90–1.22)	0.568		
Number of lifetime sexual partners				
One	ref		ref	
two to five	1.27 (1.09–1.48)	0.002	1.14 (0.96–1.36)	0.138
More than five	1.36 (1.04–1.78)	0.025	1.26 (0.92–1.74)	0.148
Condom use with regular partner				
Never	ref		ref	
Sometimes	1.38 (1.19–1.59)	< 0.001	1.19 (1.01–1.40)	0.037
Almost all the time	1.66 (1.15–2.40)	0.007	1.10 (0.71–1.69)	0.679
Always	1.22 (0.82–1.82)	0.327	0.95 (0.78–1.16)	0.634
HIV status				
Positive	ref		ref	
Negative	0.86 (0.73–1.01)	0.073	0.95 (0.78–1.16)	0.634
Unknown	0.81 (0.66–0.99)	0.05	1.07 (0.83–1.37)	0.591

Multivariable logistic regression analysis was used to get adjusted estimates for douching with any solution given the various independent variables. Women aged 35–44 years as well as those aged above 44 years were less likely to douche with any solution compared to those aged 15–24 years (AOR = 0.74; 95% CI: 0.57–0.97, *p* = 0.027 and AOR = 0.65; 95% CI: 0.49–0.87, *p* = 0.004), respectively. Women with secondary education were 1.6 times as likely to douche with any solution compared to women with no formal education (AOR = 1.64; 95%CI: 1.15–2.35, *p* = 0.007). Being in informal employment was found to reduce the odds of douching with any solution compared to being a house wife (AOR = 0.72; 95% CI: 0.58–0.89, *p* = 0.002). Odds of douching with any solution were higher among women who reported using condoms sometimes compared to their counterparts who never used condoms, although the association was weak (AOR = 1.19; 95% CI: 1.01–1.40, *p* = 0.037).

Table 3 presents findings of the association between abnormal cervical lesions and type of solution used for douching. Independent variables included age, condom use, occupation, number of sexual partners and HIV status. The prevalence of abnormal cervical lesions among women who douched with either water or any solution was 12.2% (*n* = 1447). Among women who douched with water, 12.4% had abnormal cervical lesions compared to 42.9% among those who douched with either vinegar, lemon, ginger, sugar or salt. About 10.3, 9.9 and 13.3% of women who douched with feminine wash, soap and African herbs/medicine, respectively had abnormal cervical lesions. Results from the univariate logistic regression analysis show that women who douched with solutions of either vinegar, lemon, ginger, sugar or salt were 5 times as likely to have abnormal cervical lesions compared to women who douched with water (UOR = 5.31; 95% CI: 1.19–23.75, *p* = 0.029). Douching with soap was protective against abnormal cervical lesions (UOR = 0.78; 95% CI: 0.62–0.99, *p* = 0.039). After adjusting for other independent variables, douching with either vinegar, lemon, ginger, salt or sugar was still statistically associated with abnormal cervical lesions while douching with soap was not. The risk of abnormal cervical lesions increased seven-fold in women who douched with either vinegar, lemon, ginger, salt or sugar compared to those who douched with water (AOR = 7.37; 95% CI: 1.43–38.00, *p* = 0.017).

**Table 3:** Univariate and multivariable logistic regression analysis for the association between type of douche and the risk for abnormal cervical lesions

Characteristic	UOR (95% CI)	*p*-value	AOR (95% CI)	*p*-value	Cervical Lesion Status Negative Positive n (%) n (%)	
Douche						
Plain water	ref		ref		9545 (87.63)	1348 (12.37)
Vinegar/lemon/ginger/salt/sugar	5.31 (1.19–23.75)	0.029	7.37 (1.43–38.00)	0.017	4 (57.14)	3 (42.86)
Feminine wash	0.81 (0.42–1.60)	0.539	0.52 (0.16–1.70)	0.281	87 (89.69)	10 (10.31)
Soap	0.78 (0.62–0.99)	0.039	0.78 (0.60–1.01)	0.061	744 (90.07)	82 (9.93)
African herbs/medicine	1.09 (0.38–3.13)	0.874	0.30 (0.40–2.27)	0.245	26 (86.67)	4 (13.33)
Age group						
15–24	ref		ref			
25–34	1.12 (0.94–1.34)	0.198	1.07 (0.88–1.31)	0.490		
35–44	1.13 (0.94–1.36)	0.177	1.04 (0.85–1.28)	0.700		
45+	0.79 (0.63–0.99)	0.037	0.79 (0.64–0.99)	0.045		
Condom use						
Never	ref		ref			
Sometimes	0.91 (0.81–1.03)	0.143	0.82 (0.72–0.94)	0.006		
Almost all the time	0.93 (0.66–1.31)	0.667	0.67 (0.46–0.98)	0.039		
Always	0.94 (0.68–1.31)	0.718	0.73 (0.51–1.05)	0.092		
Occupation						
Housewife	ref		ref			
Formal employment	0.94 (0.80–1.11)	0.484	0.90 (0.75–1.08)	0.262		
Informal employment	0.92 (0.80–1.06)	0.246	0.84 (0.72–0.98)	0.032		
Other	1.27 (1.07–1.51)	0.005	1.27 (1.06–1.53)	0.009		
Life partners						
One sexual partner	ref		ref			
2–5 sexual partners	1.18 (1.04–1.34)	0.010	1.12 (0.98–1.30)	0.103		
> 5 sexual partners	1.58 (1.28–1.96)	< 0.001	1.36 (1.06–1.73)	0.014		
HIV Status						
HIV+	ref		ref			
HIV-	0.54 (0.48–0.62)	< 0.001	0.52 (0.45–0.61)	< 0.001		
Unknown	0.68 (0.58–0.80)	< 0.001	0.69 (0.57–0.83)	< 0.001		

## Discussion

The current study found that vaginal douching with any solution other than water increased the risk of abnormal cervical lesions, as women who used either vinegar/lemon/ginger/salt or sugar exhibited elevated risk. Our findings are consistent with those from similar studies, albeit the other studies looked at douching in general. In a survey conducted in the United States, authors posited that douching had the potential to increase the risk of cervical cancer as the former was high-risk for HPV infection. There was a 40% higher risk of a high-risk infection in women who douched [13]. In Taiwan, post-coital vaginal douching was a risk factor for the non-regression of low-grade squamous intraepithelial lesions (LSIL) (OR = 3.14; 95% CI: 1.04–9.49) [19]. In a review of evidence to discourage douching, Cottrell [14] cites increased risk of cervical cancer as one of the serious outcomes associated with douching. A study conducted among patients with cancer of the cervix in Buffalo and Kenmore, New York, revealed a direct association between the frequency of douching and the risk of both invasive cervical cancer and carcinoma in situ [20]. Peters et al. [21] found that the “frequency-years” of douching contributed independently and significantly to the risk of invasive cervical cancer among Latinas and non-Latinas in Los Angeles County. In a meta-analysis, Zhang et al. [15] found that douching was modestly associated with cervical cancer, when they aggregated studies that looked at both invasive cervical cancer and carcinoma in situ together or at invasive cervical cancer alone (RR = 1.25, 95% CI: 0.99, 1.59). However, other studies found inconsistent results with respect to vaginal douching and cervical cancer [22–24].

An important finding of this study is that specific douches predispose women to the risk of abnormal cervical lesions. Our study found elevated risks of abnormal cervical lesions among women who used either vinegar, lemon, ginger, salt or sugar solutions for vaginal douching. Other douches had a protective effect albeit there was not enough statistical evidence to support the observed associations. Seay [25] also found an association between risk for HPV infection and specific douches. A similar observation was made by Martino et al. [6] who argued that whether or not douching had adverse effects was probably dependent on the type of solution used. Evidence showing that certain douches may interfere with the conditions suitable for the survival of lactobacilli strains and thereby compromising the epithelial cell integrity [26] could explain the increased risk for abnormal cervical lesions in our study.

The major limitation of our study is that the programmatic data that we used for investigating vaginal douching did not collect information on the frequency of douching per week or on the frequency years of engaging in the practice. As noted from some studies discussed in this paper, the risk of cervical lesions varied by the frequency and years of douching. However, we posit that the elevated risk among women who used vinegar/ginger, lemon/sugar or salt provides substantial evidence to discourage douching with these solutions among Zambian women.

## Conclusion

We find elevated risk of abnormal cervical lesions among women who use certain douches. We argue, therefore, that certain douches could potentially put women at higher risk of abnormal cervical lesions relative to water. Health promotion messaging should therefore describe the possible health risks of vaginal douching with certain solutions such as vinegar, ginger, lemon, sugar and salt. These messages should be targeted, especially at younger women, house-wives, women with secondary education and women who use condoms sometimes, in whom the practice of vaginal douching with solutions other than water is higher. There is need for further research to examine the risk of abnormal cervical lesions among women who have ever vs never douched. Future research should also take into consideration the effect of frequency as well as years of douching on risk of abnormal cervical lesions.

## Data Availability

The data that support the findings of this study are available from the Ministry of Health but restrictions apply to the availability of these data, which were used under license for the current study, and so are not publicly available. Data are however available from the authors upon reasonable request and with permission of the Ministry of Health.

## Abbreviations

AIDS: Acquired Immune Deficiency Syndrome
AOR: Adjusted Odds Ratio
CC: Cervical Cancer
CCPPZ: Cervical Cancer Prevention Programme in Zambia
CI: Confidence Interval
CIDRZ: Centre for Infectious Disease Research in Zambia
ERES: Research Ethics and Science (ERES) Converge
HIV: Human Immunodeficiency Virus
UNZA: University of Zambia
UOR: Unadjusted Odds Ratio
VIA: Visual Inspection with Acetic-acid
